# Natural transformation in Gram-negative bacteria thriving in extreme environments: from genes and genomes to proteins, structures and regulation

**DOI:** 10.1007/s00792-021-01242-z

**Published:** 2021-09-20

**Authors:** Beate Averhoff, Lennart Kirchner, Katharina Pfefferle, Deniz Yaman

**Affiliations:** Molecular Microbiology and Bioenergetics, Institute of Molecular Biosciences, Goethe University Frankfurt, Max-von-Laue-Str. 9, 60438 Frankfurt, Germany

**Keywords:** DNA uptake, *Thermus*, *Acinetobacter*, Thermophile, Desiccation resistance

## Abstract

Extremophilic prokaryotes live under harsh environmental conditions which require far-reaching cellular adaptations. The acquisition of novel genetic information via natural transformation plays an important role in bacterial adaptation. This mode of DNA transfer permits the transfer of genetic information between microorganisms of distant evolutionary lineages and even between members of different domains. This phenomenon, known as horizontal gene transfer (HGT), significantly contributes to genome plasticity over evolutionary history and is a driving force for the spread of fitness-enhancing functions including virulence genes and antibiotic resistances. In particular, HGT has played an important role for adaptation of bacteria to extreme environments. Here, we present a survey of the natural transformation systems in bacteria that live under extreme conditions: the thermophile *Thermus thermophilus* and two desiccation-resistant members of the genus *Acinetobacter* such as *Acinetobacter baylyi* and *Acinetobacter baumannii*. The latter is an opportunistic pathogen and has become a world-wide threat in health-care institutions. We highlight conserved and unique features of the DNA transporter in *Thermus* and *Acinetobacter* and present tentative models of both systems. The structure and function of both DNA transporter are described and the mechanism of DNA uptake is discussed.

## Introduction

Microbial life has been detected in virtually every environment on earth, even in extreme environments such as hydrothermal vents, salt-saturated alkaline ponds, acidic hot springs, antarctic ice, aridic soils but also in plant, animal and human hosts. To survive in such different environments, microorganisms must have evolved phenotypic traits mediating the adaptation to different environmental conditions. The adaptation of microorganisms is achieved by different mechanisms, including mutational evolution of genes, acquisition of novel genetic information even from phylogenetically unrelated organisms, intragenomic rearrangements of genetic information, gene duplications, gene loss and modification of gene expression. The increasing number of microbial ecology studies and genome analyses provide growing evidence that the acquisition of foreign genes by HGT is a major force for bacterial adaptation to changing environments (Arber 2014; Blesa et al. 2018; Brito 2021). The availability of complete genomic sequences enabling broad analyses of nucleotide composition and patterns of codon usage bias provides the opportunity to measure and to compare the cumulative amount of laterally transferred genes within distinct bacterial genomes. Theses studies revealed that large portions of bacterial genomes are attributable to horizontal transferred genes (Innamorati et al. 2020).

Three different mechanisms of HGT in bacteria have been identified: conjugation, transduction, and transformation. Among these distinct mechanisms of DNA transfer, natural transformation (natural competence), which describes the uptake and incorporation of naked DNA, allows the uptake of genetic material from diverse bacterial species and is, perhaps, the most versatile mechanism. This hypothesis is supported by the finding that the ability to take up free DNA via natural transformation is widely distributed among representatives of very different phylogenetic and trophic groups and to date, more than 80 bacterial species have been shown to undergo natural transformation (Johnston et al. 2014).

The regulatory signals triggering natural competence, the signal transduction pathways, the subunits of these macromolecular transporters and the mechanisms by which the DNA is taken up and recombines with the genome has been studied in several model organisms, such as the Gram-positive *Bacillus subtilis* and *Streptococcus pneumoniae*, and the Gram-negative *Acinetobacter baylyi*, *Neisseria gonorrhoeae*, *Haemophilus influenzae* and *Vibrio cholerae* (Averhoff and Graf 2008; Dubnau and Blokesch 2019; Johnston et al. 2014). The results from these studies led to models of DNA transporter in Gram-negative and Gram-positive bacteria.

Among the thermophilic bacteria members of the genus *Thermus*, such as *Thermus thermophilus* HB27 and *T. thermophilus* HB8, *Thermus flavus* AT62, *Thermus caldophilus* and *Thermus aquaticus* YT1 are known to exhibit high competence for natural transformation (Koyama et al. 1986). *T. thermophilus* HB27 which thrives in environments of up to 85 °C, has become a model organism to study natural transformation systems of bacteria thriving in extreme habitats. This thermophile has the most efficient DNA uptake system known so far which binds and takes up DNA from members of all domains of life and HGT has been suggested to play a major role in adaptation of *T. thermophilus* to its extreme environment (Blesa et al. 2018; Schwarzenlander and Averhoff 2006).

Among the Gram-negative bacteria, *Acinetobacter baylyi* ADP1 has served for decades as a model of genetic competence. Recent studies revealed that opportunistic human pathogenic *Acinetobacter* *baumannii* strains are also able to take up free DNA from the environment and this trait is suggested to contribute to the acquisition of new antimicrobial resistances and virulence traits (Wilharm et al. 2013). Both, *A. baylyi* and *A. baumannii* are able to thrive in extreme environments such as both exhibit an outstanding resistance to very low water activities (Antunes et al. 2011; König et al. 2020; Sand et al. 2011). Recent studies of the molecular basis of desiccation and osmostress resistance of *A. baylyi* and *A. baumannii* strains led to the identification of compatible solutes, such as mannitol, glycine-betaine, glutamate and trehalose being important for survival in environments with low water activities (Zeidler and Müller 2019). This review summarizes the current knowledge on physiology, structure and function of the transformation machineries of members of the two phylogenetically distant genera *Acinetobacter* and *Thermus*. Based on physiological, molecular, biochemical and structural data, we present models of the natural transformation machineries in *Acinetobacter* and *Thermus*.

## Physiology of natural transformation in *Acinetobacter*

Genetic competence for natural transformation has been defined as a physiological state that permits the uptake of exogenous DNA. This process can be dissected into four discrete, sequential steps, such as competence induction, DNA binding, DNA translocation across the inner (IM) and outer (OM) membrane, and recombination of the incoming DNA with homologous counterparts in the genome or plasmid regeneration. Among the Gram-negative soil bacteria *A. baylyi* ADP1 (formerly named *Acinetobacter* sp. ADP1 or BD413) was one of first representatives found to undergo natural transformation and the physiology of natural transformation of *A. baylyi* ADP1 has been under close investigation and summarized in several reviews (Averhoff and Graf 2008; Palmen and Hellingwerf 1997). These studies revealed that competence of *A. baylyi* is growth phase-dependent, such as highest transformation frequencies were found immediately after dilution into fresh medium. Competence decreases with decreasing nutrient availability; this can be taken as indication that transformation does not serve for nutrient uptake. Furthermore, natural transformation in *A. baylyi* strongly depends on divalent cations such as Ca^2+^, Mn^2+^, Mg^2+^ and requires energy. Whether the latter has to be supplied in form of ATP or the transmembrane electrochemical proton potential or both, remains to be elucidated. Transformation frequencies strongly depend on the length of the incoming DNA fragment, and an increase in fragment size from 0.3 to 3.8 kb resulted in an increase of the transformation frequencies in a biphasic manner with maximum transformation frequencies of  > 10^−1 ^transformants/viable count. The finding that fragments below 600 bp resulted in a rather inefficient transformation is attributed to an exonuclease-mediated degradation of an average of 500 bp of each incoming fragment. The finding that *A. baylyi* does not discriminate between heterologous and homologous DNA has led to a broad application of *A. baylyi* as model strain for transformation studies simulating environmental conditions.

## The natural transformation machinery in *A. baylyi*

**Fig. 1:**
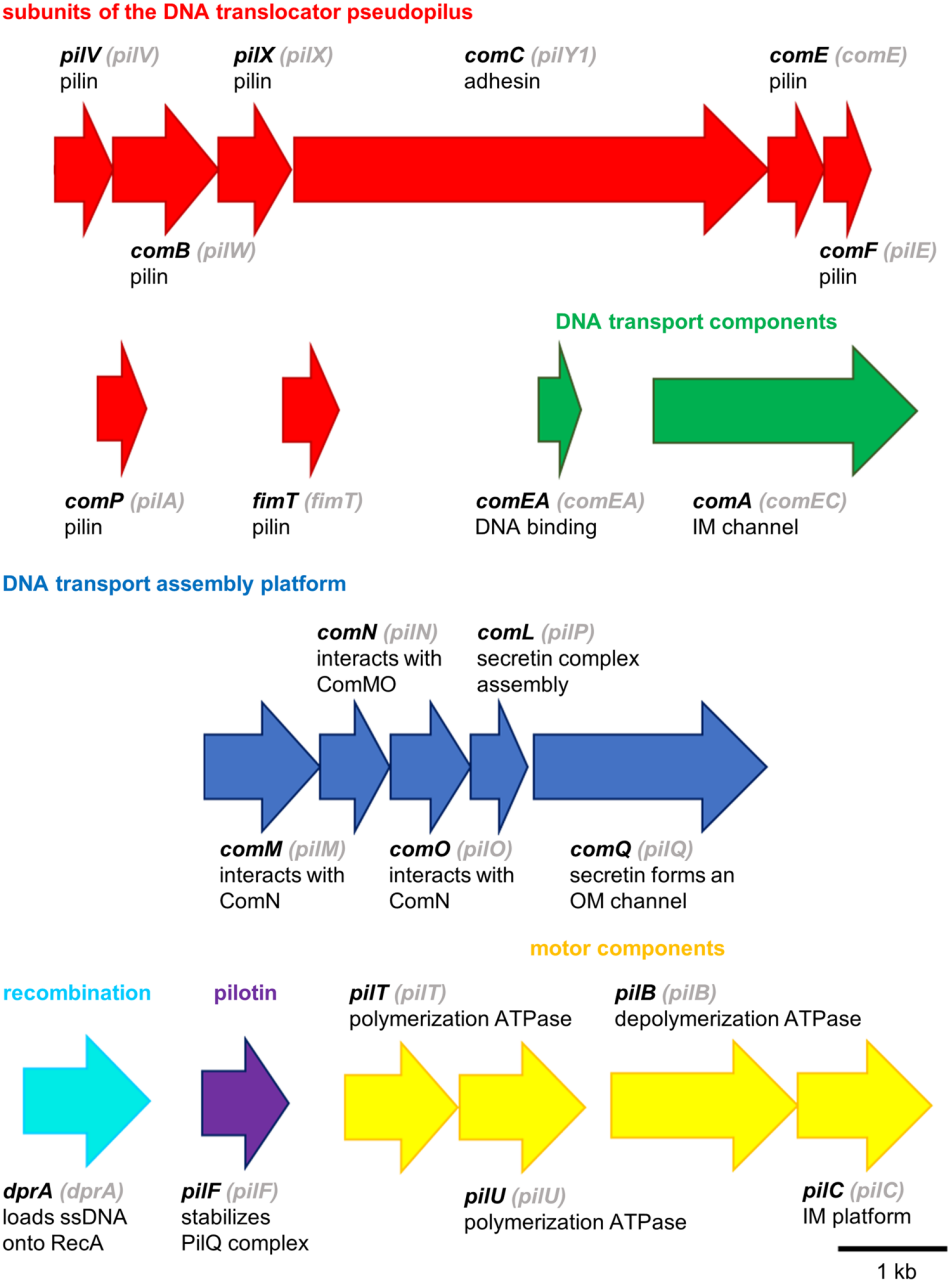
Organization and function of the competence genes in *A. baylyi* ADP1 (bold letters) and *A. baumannii* A118 (in brackets). Genes are represented by arrows indicating their approximate length and direction of transcription. The color code of the arrows reflects the general function of the proteins. The distinct function of each protein is stated underneath the gene designations

Components of the *A. baylyi* natural transformation machinery were identified by mutant studies and led to the identification of twenty one competence genes (Table 1). (Averhoff and Graf 2008; Hülter et al. 2017; Leong et al. 2017). These genes are organized in ten distinct chromosomal loci (Fig. 1).

**Table 1:** Competence genes in *A. baylyi* ADP1

Gene name	Locus tag in ADP1 (ACIADxxxx)	Gene product
*comP*	3338	Pilin
*comB*	3318	Pilin
*comE*	3315	Pilin
*comF*	3314	Pilin
*pilV*	3319	Pilin
*pilX*	3317	Pilin
*fimT*	695	Pilin
*comC*	3316	Adhesin
*pilC*	0361	Pilus assembly platform
*comM*	3360	Pilus assembly platform
*comN*	3359	Pilus assembly platform
*comO*	3357	Pilus assembly platform
*comL*	3356	Pilus assembly platform
*comQ*	3355	Secretin
*pilF*	0558	Secretin associated pilotin
*pilT*	0912	Pilus retraction ATPase
*pilU*	0911	Pilus retraction ATPase
*pilB*	0362	Pilus assembly ATPase
*dprA*	0209	DNA processing protein
*comEA*	3064	DNA binding protein
*comA*	2639	Inner membrane channel

Clues to the function of the *A. baylyi* competence proteins were derived from similarities of the competence proteins, mutant studies, immunological and biochemical analyses and are summarized in Table 1 and Fig. 1. The seven competence proteins, ComB, ComP, PilV, PilX and FimT are absolutely essential for natural transformation. These proteins are hydrophilic and are similar to pilins, the structural subunits of type IV pili (T4P). T4P have been thoroughly investigated in several pathogenic bacteria (Dos Santos Souza et al. 2020; Muñoz et al. 2020; Rehman et al. 2019); they are important virulence factors functioning as bacterial adhesins and are also involved in twitching motility (Ligthart et al. 2020). Proteins with similarities to pilins are also involved in protein secretion machineries such as Xcp’s in *Pseudomonas aeruginosa*, Pul’s in *Klebsiella oxytoca*, Out’s in *Erwinia* spp., Exe’s in *Aeromonas hydrophilia*, and Xps’s in *Xanthomonas campestris*, and prepilin-like proteins are also known to be implicated in DNA transfer systems in several Gram-positive and Gram-negative bacteria (Piepenbrink 2019).

It is important to note that the similarities of the pilins of the *A. baylyi* DNA transport machinery to pilins of T4P are only very high (up to > 90%) within the first 40 N-terminal amino-acids. The mature pilins and the pilin-like components of T4P, protein secretion systems, and DNA transfer machineries are generated by processing of pilin precursors the so called prepilins. The prepilins share a short leader peptide which is followed by an 8-amino-acid cleavage motif [KRHEQSTAG]_1_-G_2_-[FYLMIV]_3_-[ST]_4_-[LT]_5_-[LIVP]_6_-E_7_-[LIVMFWSTAG]_8_, essential for the processing by a prepilin peptidase and a pair of cysteine residues near the carboxy terminus (Strom and Lory 1991). The presence of prepilin peptidase signal motifs in the pilins of *A. baylyi* suggests that these competence proteins are directed to the IM and processed to mature pilins by a prepilin peptidase.

The function of ComP, which was the first pilin-like competence protein identified in *A. baylyi*, has already been under close investigation. DNA binding and uptake studies with *comP*-mutants revealed that ComP is absolutely essential for DNA binding and/or uptake (Porstendörfer et al. 1997). ComP was found to be glycosylated, a trait often found in extracellular proteins. However, glycosylation is not required for the function of ComP in the DNA uptake machinery (Porstendörfer et al. 2000). Immunological data clearly assigned ComP to the IM, to the periplasm and the OM. The cellular localization of ComP together with the results from the DNA binding and uptake studies and the significant similarities of ComP to pilins are strong arguments for the conclusion that ComP is part of a pilus-like shaft (pseudopilus) which extends from the IM through the periplasm and the OM and mediates DNA transport through the OM and the periplasm. This structure is probably not made of a single subunit, but might also contain the pilin-like competence proteins ComB, ComE, ComF, PilV, PilX and FimT. The actual stoichiometry of the pilin-like subunits is unknown but some hints to the structure of the suggested heterooligomer come from genetic data. The disruption of *comB, comP* and the deletion of *pilV, pilX* or *fimT* resulted in complete transformation deficiencies, whereas disruption of *comE* and *comF* led to only 10- to 1000-fold reduced transformation frequencies. Apparently, the absence of ComE or ComF can be tolerated, or ComE and ComF can be replaced, to some extent, by the other pilins. On the other hand, it should be noted that ComE and ComF cannot substitute for ComP, ComB, PilV, PilX or FimT. This suggests that ComE and ComF are accessory factors of minor importance for the pilus-like structure involved in DNA uptake.

The competence protein ComC is similar to the gonococcal T4P tip protein PilC, which is absolutely essential for DNA binding and uptake (Link et al. 1998; Rudel et al. 1995). Due to the sequence similarities and the structural characteristics together with the DNA binding and uptake studies it is conceivable that ComC is localized at the cell periphery, probably at the tip of the DNA-transporting structure, and involved in binding and transport of DNA.

The proteins described so far are most likely part of a structure anchored in the IM and spanning the periplasm and the OM. For the latter, a bushing which anchors the structure to the lipid layer of the OM, like the L-Ring protein of the flagellum, can be envisaged. The ComQ protein, which is absolutely essential for natural transformation, is suggested to fullfill this function. The ComQ protein exhibits significant similarity to members of a family referred to as secretins (Silva et al. 2020). Members of the secretin family have been shown to form highly stable oligomeric rings in the OM; these rings are the portal for pilus export or protein transport across the OM and have been identified also in many natural transformation systems (Majewski et al. 2018). Secretins are supposed to consist of two domains, a C-terminal region, comprising of four highly conserved regions shown to be important for complex formation and located in the OM and an N-terminal non-conserved region, thought to extend into the periplasm and to interact with other components of the export apparatus. The similarities of ComQ to the members of the secretin family suggest that several ComQ monomers form a multimeric ring-like structure in the OM. This might be the portal for a DNA-transporting pilus-like shaft.

The role of the competence genes *comM*, *comN*, *comO* and *comL*, which are clustered with *comQ* (Fig. 1), can be derived from their similarities to the T4P proteins PilM, PilN, PilO, and PilP, respectively. The genes of this competence cluster share their organization with the conserved T4P genes. As predicted from the sequence, ComL is probably a lipoprotein. This would place ComL also to the OM. This has also been suggested for the gonoccocal PilP, which is similar to ComL. Since the levels of multimerized gonococcal PilQ (similar to ComQ) were reduced in *pilP*-mutants, it was suggested that PilP is required for PilQ stability (Drake et al. 1997). The same could be true for ComL/ComQ in *A. baylyi*. PilT and PilU are close homologs to each other and are homologs of retraction ATPases which play a crucial role in T4P dynamics and are often also essential for T4P-linked natural transformation (Craig et al. 2019). Mutation of retraction ATPases abolishes T4P depolymerization visible by a hyperpiliation phenotype of the mutants (Graupner et al. 2001). T4P have been found to bind DNA and are suggested to bring the DNA to the cell surface followed by pulling the DNA through the OM into the periplasm via depolymerization of the pilus. The competence protein PilF exhibits significant similarities to PilF in *P. aeruginosa* and *N. gonorrhoeae* and is suggestd to act as pilotin assisting with secretin complex assembly and localization. PilC is a member of the conserved PilC/GspF family of integral membrane proteins, which are found in type II secretion systems (T2SS), T4P, archaeal flagella and natural transformation systems (Peabody et al. 2003). Studies have shown that GspF proteins interact with the IM assembly platform via PilO and with cytoplasmic motor ATPases thereby linking the ATPase-dependent energy release to pilus/pseudopilus assembly (Georgiadou et al. 2012). Whether this is also the case for PilC in *A. baylyi* remains to be shown. The *pilC* gene is preceded by a conserved *pilB* gene which encodes a T4P assembly ATPase*.* PilB is suggested to power the assembly of the DNA translocator pseudopilus.

The competence protein ComEA exhibits significant similarities to conserved DNA binding proteins in DNA transporter of Gram-positive and Gram-negative bacteria. ComEA contains a DNA-binding helix–hairpin–helix motif at the C-terminus and has DNA binding activity without detectable sequence specificity (unpublished data). These findings are similar to the findings in other transformable bacteria. ComEA in *A. baylyi* is suggested to be a periplasmic protein. This corresponds to ComEA of *V. cholerae* that plays an essential role in transport of DNA across the OM (Seitz and Blokesch 2014).

The competence protein ComA is a hydrophobic protein of M_r_ 90,500 Da. Secondary structure analyses predict at least eight transmembrane helices. ComA is similar to polytopic IM proteins of DNA transporter such as the competence protein ComA in *N. gonorrhoeae*, ComEC in *B. subtilis*, Rec-2 in *Haemophilus influenzae*, and CelB in *S. pneumoniae* (Clifton et al. 1994; Facius and Meyer 1993; Hahn et al. 1993; Pestova and Morrison 1998). Based on these findings, ComA is suggested to be involved in DNA transport through the IM. The DNA processing protein DprA initiates the integration of the incoming DNA into the chromosome by loading RecA onto ssDNA.

## Natural competence in *A. baumannii*

The soil bacterium *A. baylyi* ADP1 has been the model strain for research on *Acinetobacter* for decades. These studies led to the discovery of the β-ketoadipate pathway and its regulation, its outstanding metabolic potential, fostered its use in biotechnology, shed light onto the role of DNA strand slippage in genetic instabilty and provided significant insights into the role of natural transformation in HGT, its physiology and its the molecular details (Young et al. 2005). In recent years, another species of the genus *Acinetobacter*, *A. baumannii*, has gained considerable interest since it is an opportunistic pathogen that has become a world-wide threat in health-care institutions (Sarshar et al. 2021). *A. baumannii* is feared for its ability to acquire virulence- and antibiotic resistance traits by DNA uptake. *A. baumannii* is very successful in clinical settings, since it survives on dry surfaces, for example, furnitures, equipment or door knobs even for months. Therefore, it is a constant source of contamination and spreads from there to the patient which often leads to severe infections or even death. The finding that a plethora of *A. baumannii* strains are naturally competent suggests that natural transformation significantely contributes to the acquisition of virulence traits and antibiotic resistances.

So far, only a few studies have addressed natural transformation and its molecular basis in *A. baumannii* but the overall mechanism of DNA uptake is very similar to the DNA uptake of *A. baylyi.* Although recent studies suggest that there are differences in natural transformation dynamics and pilus regulation in *A. baumannii* (Vesel and Blokesch 2021). Interestingly, the DNA uptake of some *A. baumannii* strains depends on movement along wet surfaces, whereas others are transformable also in liquid culture (Hu et al. 2019; Wilharm et al. 2013). Based on homology, the twenty one competence genes identified in *A. baylyi* were also detected with analogous genomic organization in the transformable *A. baumannii* strain A118 (Fig. 1). Mutant studies with *A. baumannii* A118 confirmed that *pilA*, *pilQ* (secretin, *comQ* homologue), *pilT* (retraction ATPase), *comEA* (DNA-binding protein), *comF* (pilotin) and *dprA* (DNA protecting protein) are required for natural transformation, as observed in *A. baylyi* (Vesel and Blokesch 2021). Our search for conserved competence genes in the genomes of different *A. baumannii* strains revealed that the genetic composition of the DNA translocator and the organization of the genes in *A. baumannii* strains are essentialy the same. However, it has to be noted that natural transformation is not an ubiquitous trait in *A. baumannii*. Many strains, including environmental and clinical isolates have never been observed to take up free DNA (Wilharm et al. 2017). Interestingly, the genetic composition of the DNA translocator in transformable and nontransformable *A. baumannii* strains is identical. Thus, the nontransformability of some strains cannot be explained by the absence of conserved structural genes of the DNA transporter. One possibility could be that the mode of competence induction differs significantely and the induction conditions have yet not been elucidated in the nontransformable strains. Another possibility could be that some of the competence genes have lost their functionality due to mutations in the coding sequence or in the promotor regions. Another unsolved question is whether uptake of DNA is induced by growing cells on (wet) surfaces or also in planktonic cells (Hu et al. 2019); there are conflicting reports that need to be resolved in the future.

## Mechanism of DNA transport in *Acinetobacter*

**Fig. 2:**
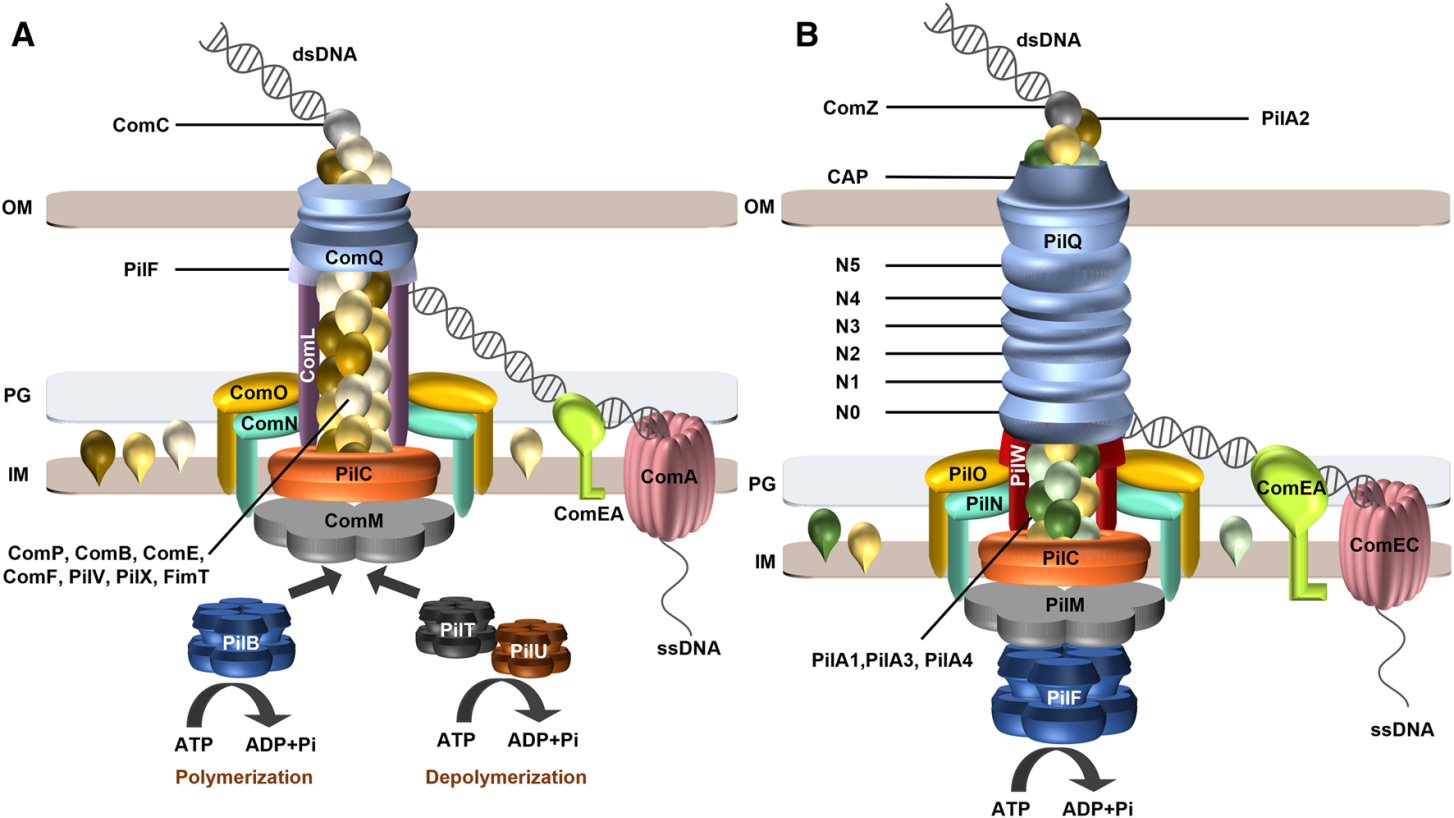
Model of DNA uptake in *A. baylyi* ADP1 (**A**) and *T. thermophilus* HB27 (**B**). Double stranded (ds) DNA binds to a DNA receptor at the tip of a pseudopilus. The pseudopili of *A. baylyli* ADP1 comprise of the pilins ComP, ComB, ComE, ComF, PilV, PilX and FimT whereas the pseudopili of *T. thermophilus* comprise of the pilins PilA1–A4 and ComZ. The tip is formed by ComC in *A. baylyi* ADP1 and ComZ and PilA2 in *T. thermophilus*. The DNA is pulled through a secretin channel in the OM comprising of multimeric secretin subunits (PilQ, ComQ) by depolymerization of the pseudopilus. The secretin of *T. thermophilus* is unique since it contains six stacked rings guiding the pseudopilus through the periplasm. ComEA binds the incoming DNA thereby supporting the transport of the DNA through the periplasm. The polytopic IM ComEC (ComA) protein forms an IM channel transporting the DNA through the IM. A DNA translocator assembly platform in the IM comprises of PilMNOW (ComMNOL). The assembly of the DNA transporter pseudopilus in *T. thermophilus* is powered by the unique polymerization ATPase PilF, which binds two second messenger c-di-GMP molecules. PilF was found to be connected to the pseudopilus in *T. thermophilus* via PilM as coupling protein. Analogously we suggest that ComM couples the assembly ATPase PilB to the pseudopilus in *A. baylyi* ADP1. Two deassembly ATPases, PilT and PilU, are suggested to mediate the depolymerization of the pseudopilus in *A. baylyi*, thereby pulling the DNA through the OM. Conserved depolymerization ATPases have not been identified in the natural transformation system in *T. thermophilus* so far. A bifunctional PilF or a non-conserved ATPase might mediate depolymerization of the pseudopili in *T. thermophilus*

Many components of the transformation machinery in *Acinetobacter* are related to structural and assembly components of T4P. Based on these similarities and together with the experimental evidence from mutant studies, we propose a hypothetical model for the DNA transport machinery, which is presented in Fig. 2A. The pilins ComP, ComB, PilV, PilX and FimT are the major structural subunits of a DNA transporter pseudopilus whereas ComF and ComE are minor pilins which are not absolutely essential for natural transformation. The finding that *comP*, *comC*, *comE* and *comF* mutants were unaffected in piliation but defective or impaired in natural transformation suggests that a short DNA translocator pseudopilus is important for DNA uptake of *A. baylyi*. In contrast, in *A. baumannii*, a role of the long pilus structures in natural transformation is discussed. This is due to the correlation of growth phase-dependent T4P biosynthesis and natural transformation and the finding that distinct regulators modulate piliation and natural transformation (Vesel and Blokesch 2021). ComC is probably located on the tip of the structure and involved in DNA binding. Double stranded DNA binds to the surface of the tip of the pseudopilus and is subsequently transported through the secretin (ComQ) channel in the OM (Fig. 2A). Retraction of the DNA translocator pseudopilus is suggested to pull the DNA into the periplasm, where it is bound to the DNA binding protein ComEA followed by the transport through the IM via an IM channel formed by the polytopic IM protein ComA. Because the DNA enters the cell as single stranded form, transport is coupled to the degradation of one DNA strand. The DNase mediating this single strand degradation has not been identified so far. Depolymerization of the pilus structures might be mediated by the depolymerization ATPase PilT and PilU, whereas polymerization might be mediated by the assembly ATPase PilB. The proteins ComM, ComN, ComO and ComL form the IM assembly platform of the DNA transporter which connects the energy generating cytoplasmic ATPases with the DNA transporter pseudopilus.

However, many questions such as the mechanistic coupling of IM and OM transport, the energetics and the molecular details of the interactions of the subunits of this multicomponent transporter spanning the cell periphery are still unanswered and have to be addressed in the future for a better understanding of the mechanism of DNA uptake.

## The natural transformation system of *T. thermophilus* HB27

The DNA translocator of *T. thermophilus* HB27 contains at least 15 components (Table 2), many of them are also essential for T4P biogenesis such as the assembly ATPase PilF which powers the DNA transporter, the IM assembly platform comprising of PilMNO, the unique PilW protein, the major pilin PilA4 and the secretin PilQ guiding the DNA through the OM (Friedrich et al. 2002, 2003; Karuppiah et al. 2013). A high resolution structure of purified PilQ complexes as well as the in situ structure of PilQ complexes in the open and closed state were unravelled (Gold et al. 2015). PilQ forms macromolecular complexes which consist of 13 protomers forming a stable cone and a cup structure and six stacked rings (N0–N5) with a large central channel (Burkhardt et al. 2011; D’Imprima et al. 2017). PilQ differs from all known secretins due to the extended N-terminal domains forming the six ring structures and two gates. The ααβαββα fold is the N0 ring-forming domain and four βαββα domains form the N2 to N5 rings (Salzer et al. 2016a). The PilQ complex is highly dynamic and undergoes major conformational changes by opening and closing of two periplasmic gates, thereby making way for pilus extrusion.

**Table 2:** Competence genes in *T. thermophilus* HB27

Gene name	Locus tag in HB27 (TT_Cxxxx)	Gene product
*pilA1*	0854	Pilin
*pilA2*	0855	Pilin
*pilA3*	0856	Pilin
*pilA4*	0858	Pilin
*comZ*	0857	Adhesin
*pilC*	0440	Pilus assembly platform
*pilM*	1013	Pilus assembly platform
*pilN*	1014	Pilus assembly platform
*pilO*	1015	Pilus assembly platform
*pilW*	1016	Pilus assembly platform
*pilQ*	1017	Secretin
*pilF*	1622	Pilus assembly ATPase
*comEA*	AF319938^a^	DNA binding protein
*comEC*	1603	Inner membrane channel
*dprA*	1873	DNA processing protein

^a^Annotation of *comEA* (TT_C1602) in the genome of *T. thermophilus* HB27 (AE017221.1) has been corrected

The localization of PilQ in the OM was found to be strictly dependent on the unique protein PilW. PilW is essential for natural transformation and piliation and yeast two-hybrid analyses revealed that PilW interacts with the N1 domain of PilQ and a combination of N0 and N1 domains of PilQ (Li et al. 2013). Recently, we reported that PilW and PilQ form heteropolymeric macromolecular complexes and that PilW is essential for stability of PilQ monomers and PilQ complexes (Yaman and Averhoff 2021). Furthermore, we found that a central disordered region in PilW is important for pilus dynamics. The PilQ complex binds the DNA in the first place and is suggested to be the counter bearing for a pilus-like DNA translocator rod and the extended T4P made by the distinct pilin subunits (Fig. 2B). A subcomplex in the IM comprising of PilMNOC forms the base for retraction and extension of the T4P structures comprising the major pilin PilA4 and the DNA translocator pseudopilus comprising PilA4 and the minor pilins PilA1–3 (Karuppiah et al. 2013; Neuhaus et al. 2020). Environmental factors, such as medium composition and growth temperature, modulate the expression of different pilins of the DNA translocator and T4P (Salzer et al. 2014b). The four pilin genes are closely associated with *comZ* which also encodes a protein essential for natural transformation. Previously, it was found that ComZ and a ComZ/PilA2 complex interact with dsDNA which led to the suggestion that PilA2 assists ComZ in forming a competence pilus tip binding DNA (Salleh et al. 2019).

PilMNO subcomplexes of the IM assembly platform bind the major pilin PilA4 (Karuppiah et al. 2013). 3D reconstructions of PilMN and PilMNO bound to PilA4 suggest that PilN drives dimerization of the PilMN complex, followed by binding of two PilO monomers causing the dissociation of PilN periplasmic domains. The latter is suggested to allow binding of the pilin PilA4 to the periplasmic domains of PilN and PilO.

The DNA transporter pseudopilus is suggested to be powered by the polymerization ATPase PilF, an unique zinc-binding hexameric ATPase complex which is also essential for T4P polymerization. The traffic ATPase PilF has been shown to form a dumbbell-like structure with two elongated stacked rings, one of which is formed by the C-terminal ATPase domains and the other by the N-terminal domains (Collins et al. 2013). PilF of *T. thermophilus* is a unique traffic ATPase, since it has an unusually long N-terminus with three “general secretory pathway II protein E, N-terminal domains” (GSPII A–C), whereas all other known traffic ATPases only contain one GSPII domain. These three GSPII domains are essential for pilus assembly and twitching motility (Kruse et al. 2018). This triplication in *T. thermophilus* might lead to higher complex stability which allows for purification of hexamers without the use of assisting protein fusions as described for purifications of other traffic ATPase complexes such as GspE from *V. cholerae* or PilB from *Myxococcus xanthus* (Bischof et al. 2016; Lu et al. 2013). Recently we demonstrated that GSPII B and GSPII C bind the second messenger molecule c-di-GMP (Keller et al. 2019; Neissner et al. 2019). This suggests that GSPII B and GSPII C are important for intramolecular signal transfer contributing to the dynamics of PilF. However, further studies are required to shed light onto the function of c-di-GMP binding to PilF. We could show that PilF interacts with components of the IM assembly platform via PilM (Kruse et al. 2019). Furthermore, PilMN and PilMNO complexes were found to stimulate PilF-mediated ATPase activity. We propose that PilM interacts with the motor ATPase PilF, thereby functioning as ATPase-pseudopilin/pilin coupling protein in the DNA translocator pseudopilus and the T4P of *T. thermophilus*. Interestingly, two retraction ATPases both essential for T4P retraction are not required for the transformation machinery in *T. thermophilus* (Salzer et al. 2016b). In addition to the pseudopilus the IM anchored ComEA protein which binds dsDNA is important for the transport of DNA into a DNase-resistant state by pulling the DNA through the OM. The ssDNA is subsequently transported across the IM through a channel formed by the polytopic IM protein ComEC (ComA homologue). Interestingly, ComEC was also found to modulate transcriptional regulation of DNA transporter and T4P components (Salzer et al. 2016b). In the cytoplasm the ssDNA is supposed to be bound by DprA followed by a RecA mediated recombination with chromosomal or plasmid DNA.

Taken together, protein–protein interaction studies, structural analyses of purified proteins and subcomplexes as well as analyses of the in situ architecture suggest that the IM and OM proteins of the DNA translocator and of T4P in *T. thermophilus* form an integrated structure extending from the cytoplasm to the OM and led to a model of DNA uptake in *T. thermophilus* (Fig. 2B): DNA is bound to the secretin (PilQ) complex or to a thus far unknown DNA binding protein close to the PilQ complex in the outermost cell layer, which is comprised of an S-layer and lipids. Subsequently, the DNA is bound to a DNA translocator pseudopilus comprising of the pilins PilA1–4 and ComZ which extends into the secretin channel made up of six staggered rings. The depolymerization ATPase PilF powers retraction of the pseudopilus contributing to the transfer of the incoming DNA through the secretin channel. The incoming DNA is transferred to the DNA-binding protein ComEA which delivers the DNA to the ComEC channel mediating the transport of the DNA through the IM.

The similarities of *Thermus* competence proteins to proteins of the transformation machineries in mesophilic bacteria suggest that despite their phylogenetical distance and despite their very different habitats the extremophile *T. thermophilus* HB27 and the mesophile Gram-negative *A. baylyi* and *A. baumannii*. share structural similarities within their transformation machineries.

## Regulation of competence development in *Acinetobacter* and *T. thermophilus* HB27

Physiological studies revealed that competence development in *A. baylyi* is a highly regulated process with highest frequencies directly after transferring a stationary phase culture to fresh media (Palmen et al. 1994). Transcriptional studies confirmed these data. ComP expression gradually decreased during prolonged exponential growth, and minimal levels were detected in the middle of the logarithmic growth phase, but levels increased thereafter (Porstendörfer et al. 2000). Transcriptional studies performed with the competence genes *comB* and *comA* gave the same results (Friedrich et al. 2001; Herzberg et al. 2000). This indicates a coordinated activation of competence proteins and suggests the presence of a competence regulon. In *A. baumannii* the two-component system PilSR and the chemosensory system Pil-Chp were shown to be essential for natural transformation and T4P-mediated twitching motility (Vesel and Blokesch 2021). In *A. baylyi*, a regulatory system regulating the expression of the DNA transporter genes has not been identified so far. However, it is interesting to note that the increase in transcription of competence genes in *A. baylyi* during log phase is not paralleled by an increase in the transformation frequency. Instead, maximal competence is observed immediately after the transition from lag to the log phase and gradually declines thereafter to minimal transformation frequencies in stationary phase, suggesting that the DNA transformation machinery is synthesized prior to induction of maximal competence (Porstendörfer et al. 2000). This would guarantee a very fast activation of the DNA-uptake machinery, probably not requiring protein synthesis.

Transformation of *A. baumannii* is also observed only in a small window during growth. However it has to be noted that highest transformation frequencies of *A. baumannii* were detected in a tight window (starting 90 min after inoculation) in the exponential growth phase (Vesel and Blokesch 2021). The highest transformation frequencies of *A. baumannii* correlate with maximum transcript levels of the major pilin subunit PilA. A decrease of expression levels was observed for some of the competence proteins throughout prolonged growth, but in contrast to the transcription of competence genes in *A. baylyi* no increase starting in the middle of the expontial phase was observed in *A. baumannii*. However, the variations in expression levels have to be seen with caution since levels for “zero” were not reported and the first data point at 60 min is already at the maximum.

In contrast, relatively little is known about the regulation of competence development in *T. thermophilus* HB27 but it has to be noted that *T. thermophilus* exhibits the highest natural transformation frequencies known to date such as the entire population of *T. thermophilus* HB27 is competent (Hidaka et al. 1994). The transformation frequencies of *A. baylyi* and *A. baumannii* strains are much lower, in the range of 10^–2^–10^–7^ depending on the strain and transformation conditions. The question whether there is a growth phase-dependent difference in DNA uptake in *T. thermophilus* HB27 can not be answered yet, but is currently under investigation in our lab.

## Natural transformation machineries and T4P: distinct systems involving conserved components or linked machineries with common components?

The DNA uptake systems of naturally transformable bacteria, in general, are similar to T4P and, therefore, one of the most fundamental questions is whether the T4P present on the cell surface of transformable bacteria are involved in DNA uptake. The question has been addressed in many transformable bacteria but still the connection between pili and natural competence is not clear, and it seems that there are different answers to this question. The DNA transformation system in *B. subtilis*, which is an intensively studied model system of DNA transport in Gram-positive bacteria, was found to contain components similar to proteins implicated in T4P biogenesis (Dubnau and Blokesch 2019). However, T4P have never beeen observed in *B. subtilis.* In *B. subtilis*, a short DNA uptake pilus encoded by seven *comG* genes is important for the transport of DNA across the cell wall (Hahn et al. 1993).

In the pathogenic Gram-negative bacterium *N. gonorrhoeae*, which exhibits T4P, the structural subunit of T4P (PilE) and the T4P factor PilC are essential for DNA transformation (Fussenegger et al. 1997; Rudel et al. 1995). However, distinct *pilE* mutations were found to prevent pilus formation but did not abolish transformation competence, which suggests that the extended pilus fibre is not required for natural transformation (Obergfell and Seifert 2016). In contrast mutation of the major T4P subunit *pilE* in the pathogenic *Legionella pneumophila* and *pilA* in *P. stutzeri* abolished both, expression of T4P and competence for natural transformation (Graupner et al. 2000; Stone and Kwaik 1999). Moreover, recent studies of regulation of T4P biogenesis and natural transformation of *A. baumanii* revealed that pilus production is growth phase dependent and essential for natural transformation (Vesel and Blokesch 2021).

The pathogen *V. cholerae* exhibits three different T4P systems. One, the toxin co-regulated pilus is important for binding of the CTXΦ bacteriophage which encodes the cholera toxins and plays an important role in infection of the human host (Waldor and Mekalanos 1996). The second T4P system [mannose-sensitive haemagglutinin (MSHA) pili] is important for biofilm formation (Chiavelli et al. 2001). The third T4P system represents the chitin-regulated DNA-uptake pilus (Meibom et al. 2005). However, the pilus structure itself is obviously not required for transformation (Seitz and Blokesch 2014).

With respect to linkage between piliation and transformation in *A. baylyi* ADP1 it has to be pointed out that it possesses two types of pili, thin ones with a diameter of 3.5 nm appearing in bundles and thicker individual pili with a diameter of about 6 nm. The production of these two types of pili has already been reported in different *Acinetobacter calcoaceticus* and *A. baumannii* strains (Henrichsen and Blom 1975; Wilharm et al. 2013). Electron microscopic analyses revealed that the thin pili are important for the adherence to hydrocarbons, plastic surfaces, for agglutination of the cells and binding to red blood cells whereas the thick pili mediate a special kind of surface translocation, termed twitching motility (Gohl et al. 2006; Harding et al. 2013). In an *A. baumannii pilT* mutant, the amount of thick pili increased (Wilharm et al. 2013). Electron microscopic analyses of transformation-deficient *A. baylyi* ADP1 *comP*, *comE*, *comF* and *comC* mutants revealed that all mutants still exhibited an unaltered piliation phenotype. Moreover, twitching motility analyses of these mutants clearly showed that none of the transformation mutants is impaired in twitching motility. These findings strongly suggest that the pilus structures, as revealed by electron microscopy, are not part of a DNA binding or uptake machinery in *A. baylyi* ADP1. This would argue for the presence of two related but genetically distinct structures with different functions, which arose from a common ancestor. In contrast, inactivation of *comP* and other core components of the DNA transporter abolished and mutation of *comC* decreased twitching motility (Link et al. 1998; Porstendörfer et al. 2000). Further studies are required to elucidate the role of T4P and pilus dynamics in natural transformation of *A. baylyi*.

Functional analyses of the T4P assembly ATPase PilF in *T. thermophilus* revealed a *pilFΔGSPII* mutant strongly reduced in piliation but hypertransformable (Kruse et al. 2018). This finding also suggests that the pilus structures are not involved in natural transformation. This is also supported by the finding that mutants defective in the T4P retraction ATPases PilT1 and PilT2 had no defect in natural transformation (Salzer et al. 2014a). Furthermore, deletion of distinct ring structures in the secretin complex had a dramatic effect on piliation and T4P-mediated twitching motility but no effect on natural transformation. This is consistent with the conclusion that DNA uptake of *T. thermophilus* is independent of T4P.

Taken together, the data support the conclusion that T4P and DNA transformation are linked but the role of T4P appendages in natural transformation obviously differs in bacteria.

## Conclusions

From the similarities of the competence proteins to T4P biogenesis and protein secretion, it is obvious that these structures have evolved from a common ancestor, but differentiated into similar structures with different functions. The common property is an interaction of these structures with macromolecular structures, i. e. proteins or lipids during adhesion or DNA and protein transport. Whereas the former property does not seem to require special adaptations, the latter has to aquire a structural basis for selectivity, such as for transport of macromolecules through protein complexes in the IM and OM, the interior has to be wide enough to allow passage of the macromolecule. Transport has to be specific, at least for protein or DNA. This specificity could be brought about by specific interactions of the pilus-like structures with the macromolecules or by charge recognition. To elucidate the differences in specificity of apparently structurally similar systems is a challenge for future studies.
